# The ArfGEF GBF-1 Is Required for ER Structure, Secretion and Endocytic Transport in *C. elegans*

**DOI:** 10.1371/journal.pone.0067076

**Published:** 2013-06-19

**Authors:** Karin B. Ackema, Ursula Sauder, Jachen A. Solinger, Anne Spang

**Affiliations:** 1 Growth and Development, Biozentrum, University of Basel, Basel, Switzerland; 2 Microscopy Center, Biozentrum, University of Basel, Basel, Switzerland; Institut Curie, France

## Abstract

Small GTPases of the Sar/Arf family are essential to generate transport containers that mediate communication between organelles of the secretory pathway. Guanine nucleotide exchange factor (GEFs) activate the small GTPases and help their anchorage in the membrane. Thus, GEFs in a way temporally and spatially control Sar1/Arf1 GTPase activation. We investigated the role of the ArfGEF GBF-1 in *C. elegans* oocytes and intestinal epithelial cells. GBF-1 localizes to the cis-Golgi and is part of the t-ER-Golgi elements. GBF-1 is required for secretion and Golgi integrity. In addition, *gbf-1(RNAi)* causes the ER reticular structure to become dispersed, without destroying ER exit sites (ERES) because the ERES protein SEC-16 was still localized in distinct punctae at t-ER-Golgi units. Moreover, GBF-1 plays a role in receptor-mediated endocytosis in oocytes, without affecting recycling pathways. We find that both the yolk receptor RME-2 and the recycling endosome-associated RAB-11 localize similarly in control and *gbf-1(RNAi)* oocytes. While RAB5-positive early endosomes appear to be less prominent and the RAB-5 levels are reduced by *gbf-1(RNAi)* in the intestine, RAB-7-positive late endosomes were more abundant and formed aggregates and tubular structures. Our data suggest a role for GBF-1 in ER structure and endosomal traffic.

## Introduction

Intracellular communication between organelles along the secretory pathway is maintained by transport vesicles that bud off from the donor compartment and fuse with the target compartment. The first step of vesicle formation requires the activation of a small GTPase of the Arf/Sar family. This activation is performed by guanine nucleotide exchange factors (GEFs). While there is only one GEF for Sar1 at the endoplasmic reticulum (ER), Arf family GTPases have a number of different GEFs, consistent with number of different Arf and Arf-like proteins and the various cellular places at which these small GTPases need to be activated. All ArfGEFs contain a common SEC7 domain, which carries the exchange activity. Sec7 is the archetypal ArfGEF, first identified in yeast, which activates Arf proteins at the trans-Golgi [1] [2]. Its *C. elegans* homologue AGEF-1 has been shown to be essential for Golgi structure maintenance and transport to the plasma membrane [3]. The ArfGEFs Gea1 and Gea2 in yeast have been shown to perform overlapping functions in the activation of Arf1 for retrograde transport from the Golgi to the ER, and they presumably also act in intra-Golgi transport [4]–[6]. Their mammalian counterpart GBF1 is localized to the Golgi apparatus and has been shown to be essential for maintaining Golgi structure and traffic through the Golgi [5], [7]–[11].

Mutants in the Drosophila homologue Gartenzwerg (garz) have secretion defects, which culminate in a failure in tracheal and salivary gland development [12]–[14]. In addition, the endocytosis of GPI-anchored proteins through the pinocytic GEEC pathway requires GBF1/garz function at the plasma membrane to recruit and activate Arf1 in mammalian and drosophila cells [15], [16]. *C. elegans* PHI-34 is the closest homolog to mammalian GBF1 with 38% identity and therefore it is also referred to as GBF-1, despite of the absence of any functional data so far.

Here we analyzed the function of *C. elegans* PHI-34/GBF-1 and found that it is localized to the cis-Golgi at transitional ER-Golgi elements. In addition, GBF-1 is required for Golgi maintenance and knockdown of GBF-1 causes a strong defect in ER structure and reduction in secretion, consistent with faulty retrograde transport. We also detected defects in endocytosis in *gbf-1(RNAi)* animals. However, the defect was not obvious at the plasma membrane but rather affected early and more strongly late endosomes. Therefore, we identify a novel role for GBF-1 at endosomes.

## Materials and Methods

### General methods and strains

*C. elegans* was cultured and maintained as described previously [17] at 20°C. Bristol N2 was used as wild-type control. Strains used in this study were WHO351 (ojIs37 [*GFP::ugtp-1, unc119(+)*]) [18], RT163 (pwIs33[P*pie-1*::GFP::*rab-11*, unc-119(+)]) [19], WH327 (unc-119(ed3) III, ojIs23[GFP::SP12 unc119(+)]) [20], AZ212 (unc-119(ed3) III ruIs32[unc-119(+) pie-1::GFP::H2B]) [21], RT408 (unc-119(ed3) III pwIs116 [rme-2::RME-2-GFP]) [22], RT688 (pwIs28[pie-1p-cav-1::GFP7 + unc-119]) [3], DH1033 (sqt-1(sc103) II; bIs1[vit-2::GFP + rol-6(su1006)]X) [23]. FA086 (pwIs429[vha-6::mCherry-rab-7]; pwIs72[vha6p::GFP::rab-5 + unc-119(+)]) was made by crossing *pwIs429* [15] and pwIs72 (RT327) [24].

### qPCR

Worms fed on RNAi from L1 were harvested after 3 days at 20°C by washing them of the plate with M9 buffer [25] and allowing them to settle on the bottom of the tube. After 2 washes with M9, the worms were dissolved in Trizol (Invitrogen). RNA was extracted according to the manufacturer's instructions. The RNA was DNase digested and cleaned up on a RNA Clean & Concentrator™-5 column (Zymo Research). 700 ng of RNA was reverse transcribed using superRT reverse transcriptase (HT Biotechnology), random hexamers (Applied Biosystems), human placental ribonuclease inhibitor (HT Biotechnology) and 1 mM dNTPs (HT Biotechnology) according to the manufacturer's instructions (HT Biotechnology, UK). PCR was performed using the SyBr Fast Kit (Kapa Biosystems) according to standard recommendations in a Corbett Research RG-6000A instrument in running under Rotor-Gene software version 1.7. agef-1 and gbf-1 expression levels were normalized to pmp-3 and cdc-42 [26] using a geometric mean of their level of expression [27]. Fold differences were calculated using the delta-delta Ct method, corrected for PCR efficiency [28]. Primer sequences: agef1_F 5′-GGAACTGGACTTAATTCTGC-3′, agef1_R 5′-TTCACCGAGAGCATCTTG-3′, gbf1_F 5′-AGCAAACTGTTGGAGGAGAAG-3′, gbf1_R 5′-ACCATTGATTCAGTATTGTGAC-3′, cdc42_F 5′-CCTCTATCGTATCCACAG-3′, cdc42_R 5′-GGTCTTTGAGCAATGATG-3′, pmp3_F 5′-TCGAGAAGCTGTAGATGAGGTAC-3′, pmp3_R 5′-CTCTATGACGACGTTTCACCTG-3′.

### RNAi experiments

RNAi was performed as described [29]. For RNAi feeding experiments, plasmid L4440, containing agef-1 (Y6B3A.1) and gbf-1 (C24H11.7), sequences was retrieved from the Ahringer library and sequenced to confirm their identity [29]. NGM plates containing 1 mM IPTG and 25 µg/ml carbenicillin were inoculated with RNAi bacteria and induced for ∼12 hours at room temperature. Eggs or larvae were cultured at 20°C until adulthood.

### Imaging

For each imaging experiment we compared worms derived from the same parental plate before exposing them to RNAi. All images within an experiment were taken with identical laser intensities, gain settings, exposure, zoom and magnification.

For live epifluorescence imaging of RME-2::GFP, GFP::H2B, VIT-2::GFP and CAV-1::GFP in adult hermaphrodites or eggs a Zeiss Axioplan 2 microscope equipped with a Zeiss Axio Cam MRm camera (Carl Zeiss, Germany) was employed. Zeiss Axiovision 3.1 to 4.8 software was used to control hardware and to acquire images. For live imaging of adults, the worms were mounted on an agarose pad in M9 with 1 mM levamisole. For the imaging of eggs, adult worms where cut in egg buffer [30] with 1 mM levamisole to release the eggs. Embryos were imaged using a hanging drop method on a 12-well microscope slide (Thermo Scientific) [31]. RME-2::GFP, GFP::H2B and CAV-1::GFP and freshly hatched larvae were imaged with a Plan Apochromat 63x/NA1.40 oil DIC objective. VIT-2::GFP on whole worms was imaged with a Plan NeoFluar 10x/0.30 air objective and a Plan NeoFluar 20x/0.50 air objective. For live imaging we routinely analyzed between 10 and 20 worms of each RNAi construct per experiment. The data were collected from at least 3 independent experiments.

Fixed samples were mounted on uncoated slides in anti-fade reagent CitiFluor (Citifluor Ltd., UK). Anti-HDEL single staining of ER in oocytes and live embryo imaging of SP12::GFP and VIT-2::GFP were captured on a Andor Revolution spinning disk confocal system (Andor Technologies, Northern Ireland) mounted onto an IX2-UCB inverted microscope (Olympus, Center Valley, PA), equipped with iXon^EM^+ 885 CCD camera (Andor Technologies) and Yokogawa CSU10 Scanner Unit. A Plan Apo N 60x/1.42 oil objective was used.

All other immunohistochemistry was imaged on a Leica SP5 confocal microscope with a 100x oil objective (Leica Microsystems, Germany), 1x digital zoom at a resolution of 1024×1024 pixels. Images were acquired with LAS AF and exported as TIFF images. ImageJ was used for post-processing and analysis of all images. Each image within an experiment was treated identical with respect to brightness and contrast adjustments.

To assemble multi-image pictures of whole-mount worms, we captured bright field images on the Zeiss Axioplan 2 microscope with the Plan NeoFluar 20X/0.50 air objective. The Adobe Photoshop Photomerge automation was used for the assembly of the individual images.

Images of worm plates were taken on Zeiss stemi2000 microscope with a Nikon Coolpix 995 camera with ocular adaptor.

### GBF peptide antibody purification

Peptide antibodies against *C. elegans* GBF-1 were raised by Eurogentec (Belgium) in rabbits using peptides CCPISAGDEADSESEGG and IVLRSNRHAPSTELP. Serum was affinity-purified, using peptides coupled to an NHS-activated sepharose column and eluted with 0.2 M glycine.

### Immunoblot

Synchronized adult worms were washed from the RNAi plate with M9 buffer. Equal volume worm pellets were suspended in 2x Laemmli buffer [32] and ground with a micro-mortar and pestle (Radnoti). For analysis by SDS-PAGE and immunoblotting 20 µl of the extracts was used. Ponceau staining confirmed equal loading.

### Immunohistochemistry

Immuno-labeling was based on established protocols [30] with small adaptations. The worms were cut in egg buffer with 1 mM levamisole and transferred to a 2 ml reaction tube containing ice-cold fixation buffer (2% w/v PFA freshly dissolved in egg buffer) and fixed for 1 h on ice. The carcasses were washed and permeabilized as described [30]. Primary antibodies were incubated O/N at 4°C with rocking. Secondary antibodies were incubated for two hours at RT with rocking. Triple GFP/SEC-16/GBF-1 staining was done with additional modifications. After fixation, the gonads were first blocked in PTB (1% BSA, 1x PBS, 0.1% Tween20, 1 mM EDTA and 0.05% sodium azide) for 2 h, followed by an additional 2 h block in BLOCK (2% BSA, 2% gelatin, 2% milk powder, 0.05% Tween20 in PBS) [30], [33]. The secondary antibodies were diluted in BLOCK and incubated at RT for 2 h with rocking. After washing, the GBF-1^Alexa 647^ antibody was incubated O/N at 4°C with rocking in 10% BLOCK in PTB.

Gonads were mounted in citifluor (Citifluor Ltd., UK) on uncoated slides. The antibodies were diluted as follows: rabbit anti-GBF-1 1:25, mouse anti-GFP (Roche) 1:100, rabbit anti-GFP (Torrey Pines Biolabs, Secaucus, NJ, USA) 1:100, goat anti-mouse Alexa Fluor^®^ 594 (Molecular Probes, Life Technologies Corporation) 1:1000, goat anti-rabbit Alexa Fluor^®^ 594 (Molecular Probes, Life Technologies Corporation) 1:1000, goat anti-mouse Alexa Fluor^®^ 488 (Molecular Probes, Life Technologies Corporation) 1:1000, chicken anti-rabbit Alexa Fluor^®^ 488 (Molecular Probes, Life Technologies Corporation) 1:1000, mouse monoclonal anti-HDEL antibodies 1:25 [20] and rabbit anti-SEC16 1:1000 [34].

Direct labeled rabbit anti-GBF-1 was made by using the Alexa Fluor^®^ 647 Monoclonal Antibody Labeling Kit (Molecular Probes, Life Technologies Corporation). Manufacturer's instructions were followed. The antibody was used in a dilution of 1:100.

All antibody stainings were first optimized for consistent antibody labeling. For immunofluorescence experiments we routinely mounted 50–100 worms on a slide for analysis. The experiments were performed at least three times using comparable experimental conditions.

### Transmission Electron Microscopy (TEM)

Adult worms were synchronized by bleaching as described [35]. Late L1 offspring was fed with RNAi until adulthood. The worms were placed in egg buffer with 1 mM levamisole, and the head was cut off to release the gonads. Buffer was replaced by ice-cold pre-fixation buffer (2% w/v PFA freshly dissolved in egg buffer [30]) and fixed on ice for 20 min. After this pre-fixation, we separated the gonads from the carcasses. The pre-fixation buffer was replaced by final fixation buffer (2.5% glutaraldehyde, 1% w/v formaldehyde in 0.1 M sucrose and 10 mM PBS pH 7.4) for O/N fixation. The TEM protocol was performed as described [36]. Pictures were taken on a Philips Morgagni 80 KV microscope (Eindhoven, The Netherlands).

## Results

### Reduction of GBF-1 results in strong developmental defects and a weakened cuticle

**Figure 1:**
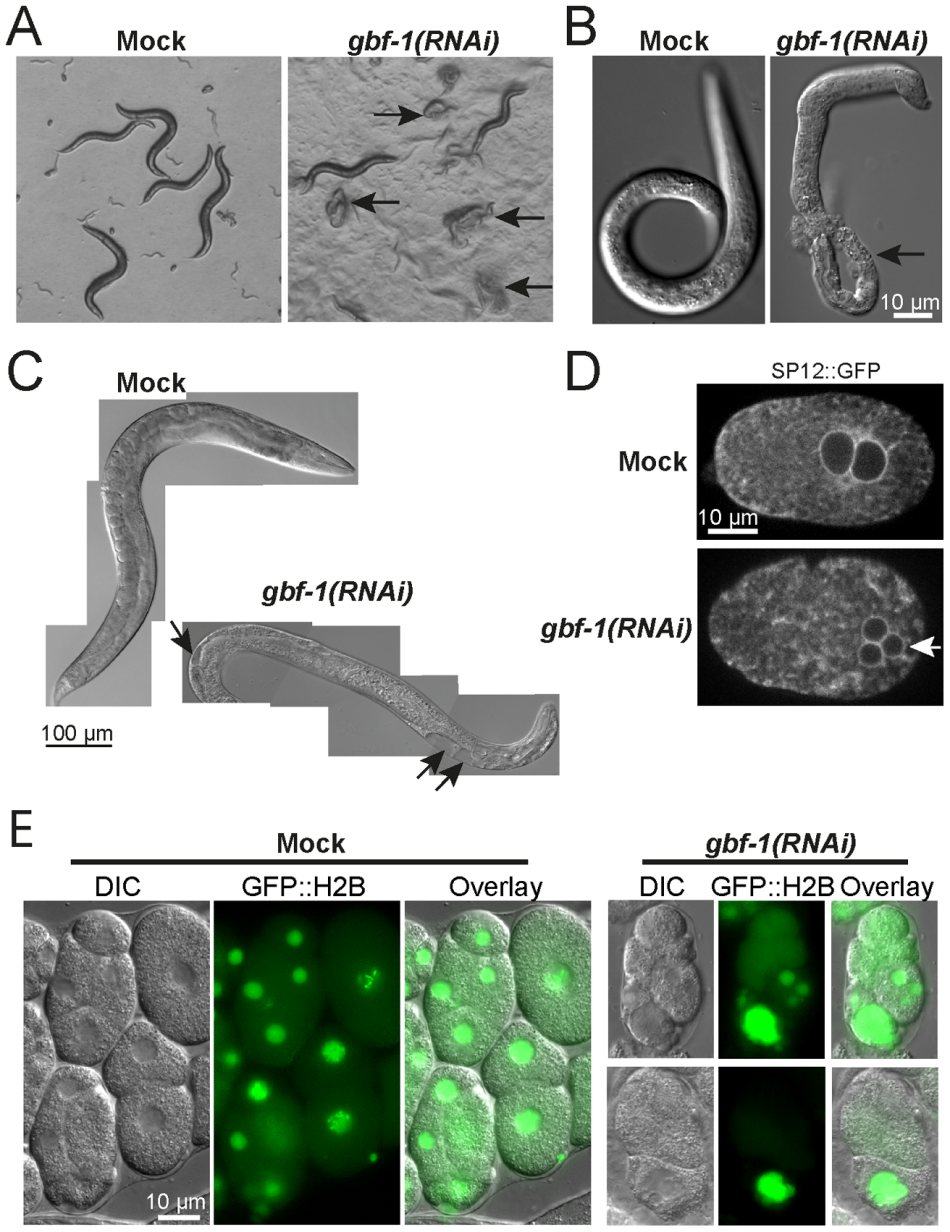
*gbf-1(RNAi)* worms causes multiple development defects. (A) *gbf-1(RNAi)* worms have a weakened cuticle. When fed from hatching on *gbf-1(RNAi)* bacteria, the majority of the worms died of cuticle bursting upon reaching adulthood. Adult worms and their offspring on agar plates. Black arrows point to worms that have bursted. This phenotype is consistently seen in all *gbf-1(RNAi)* feedings from hatching (B) Although most embryos arrested early in development in *gfb-1(RNAi)* worms, few larvae could hatch from the eggs. These larvae often displayed severe problems with their cuticle formation. Black arrow points to an intestine that is directly exposed to the environment instead of being protected by the cuticle. (C) Worms fed on *gbf-1(RNAi)* from hatching frequently accumulate liquid filled vacuoles in the body cavity. Furthermore they are sterile (no eggs in the uterus) and their intestine appears ‘clear’. Merged bright field images of whole worms. Black arrows point to vacuoles. (D) During meiosis the egg normally expels two polar bodies during anaphase I and II. In *gbf-1(RNAi)* eggs, the extrusion of the second polar body during anaphase II often failed, which can be seen by the presence of 3 nuclei (arrow) in fertilized eggs at pronuclear meeting. This phenotype was consistently seen throughout different experiments involving embryos. We used SP12::GFP marking the ER that nicely outlined the nuclei. Anterior in both one-cell embryos is on the left. A single plane confocal image is shown. (E) Offspring from worms fed on *gbf-1(RNAi)* from L3 frequently show problems with cytokinesis which results in unequal distribution of the DNA between cells. GFP::H2B was used to visualize the DNA. The experiment was performed at least three times.

We first aimed to establish the phenotypes of GBF-1/PHI-34 reduction during development. Since the null mutant in gbf-1 is lethal (National Bioresource Project Japan), we studied the function of GBF-1 in *C. elegans* by using RNAi. Feeding *gbf-1* RNAi producing bacteria from L1 stage caused cuticle rupture in the majority of the worms upon reaching adulthood, resulting in a 50 to 80% mortality rate. Moreover, the knockdown caused sterility due to embryonic lethality in the escapers that were able to reach adulthood without bursting (Fig. 1A). Freshly hatched larvae also frequently showed severe abnormalities in their cuticle formation (Fig. 1B).

These cuticle defects that lead to rupture of bursting through the vulva were never observed in *agef-1(RNAi)* worms; AGEF-1 is the homologue of yeast Sec7 [3]. Moreover, no gross morphological abnormalities in *agef-1(RNAi)* animals were detected when fed from L1 stage (data not shown). In contrast, *gbf-1(RNAi)* worms accumulated vacuole-like structures in the body cavity in more than 90% of the surviving worms (Fig. 1C). Since we were particularly interested in the function of GBF-1 in oocytes and early embryos, we decided to feed from either the L3 or L4 larval stage for maximal 48 hours. The reduced knockdown of GBF-1 allowed normal production and fertilization of oocytes. Nevertheless this treatment caused an embryonic lethal effect in 60 to 70% of the fertilized oocytes. The arrested eggs of *gbf-1(RNAi)* were easily recognized as they were generally highly distorted. We found them to be stretched, rounded-up, and leaking through the egg shell (not shown). This completely disintegrated egg shell resulted in an amorphous mass of cells and membranes in the gonad (Fig. S3B). We also observed defects in polar body extrusion in around 30% of the fertilized oocytes (Fig. 1D). In addition, in a similar percentage of eggs cytokinesis was defective (Fig. 1D).

We assessed the knockdown efficiency by qPCR, which showed an about 50% reduction of mRNA for either *gbf-1* and *agef-1* ( Fig. S1A). We successfully raised peptide antibodies against GBF-1 and observed a clear reduction of the GBF-1 signal in both immunoblot and in immunofluorescence of oocytes and intestine from *gbf-1(RNAi)* animals (Fig. S1B and C). Unfortunately, we could not raise specific peptide antibodies against AGEF-1, and GFP fusions to AGEF-1 were not tolerated by the worm.

Taken together, reduction of GBF-1 expression caused severe developmental problems starting already at fertilization. The severity and onset of the phenotype are most likely inversely correlated to the concentration of GBF-1 in the cell.

### GBF-1 is important for Golgi integrity

Next, we aimed to determine whether GBF-1/PHI-34 in *C. elegans* is indeed the bona fide homologue of mammalian GBF1, drosophila garz and yeast Gea1/2. In those systems, GBF1 was shown to be localized to the cis-Golgi [5], [9], [13].

Like in several other invertebrate cell types, the Golgi in the *C. elegans* germ line is not organized in a typical juxtanuclear Golgi ribbon, but rather in small mini stacks localized next to ER-exit sites (ERES) [18], [34], [37], [38].

**Figure 2:**
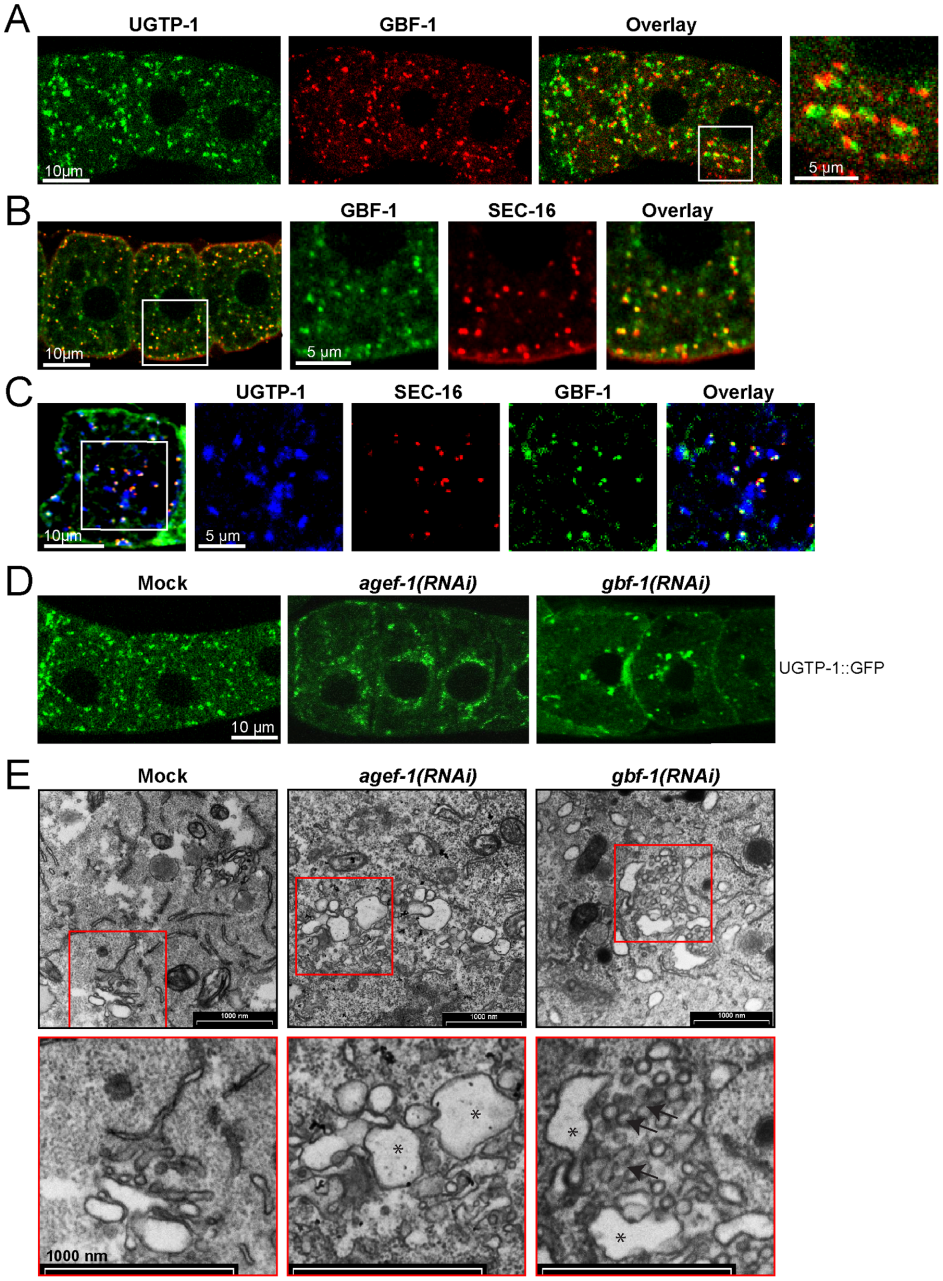
GBF-1 is important for Golgi integrity. (A) GBF-1 localizes to the Golgi in oocytes. Displayed are the three most proximal oocytes. GBF-1 partially co-localized with the medial Golgi marker UGTP-1::GFP. The third panel shows an overlay of the two channels and an enlargement of the white box is shown on the right. (B) GBF-1 is part of the Golgi t-ER region in oocytes. GBF-1 was localized juxtaposed to the ERES marker SEC-16. (C) The Golgi in *C. elegans* germ line is organized in small mini stacks localized in close proximity to ER-exit sites (ERES). GBF-1 was found ‘sandwiched’ between the Golgi marker UGTP::GFP and ERES marker SEC-16. (D) *agef-1(RNAi)* and *gbf-1(RNAi)* causes the collapse of the Golgi around the nuclear membrane and cell periphery in oocytes. A single confocal plane in the center of the cell is shown. (A–D) The orientation of the gonad is identical in all panels: the spermatheca and the most mature oocyte are located to the left. All channels are in false colors. A single confocal plane is shown. (E) Transmission electron microscopy images of oocytes. The top panel shows a part of the oocytes. The bottom panel shows an enlargement of a Golgi stack as indicated by a red box. Largely inflated cisternae were observed in both *agef-1(RNAi)* and *gbf-1(RNAi)* cells as indicated by asterisks. *gbf-1(RNAi)* Golgi stacks accumulated a lot of small-sized vesicles as indicated by black arrows. (A–E) The experiments have been performed at least three times with comparable experimental conditions using different RNAi feeding batches. The selected images are representative for the observed phenotype.

In mature oocytes, GBF-1 localization is closely associated and partially overlapping with the Golgi marker GFP::UGTP-1 [18] (Fig. 2A) and was always found in close proximity to ERES marker SEC-16 [34], confirming cis-Golgi localization (Fig. 2C). Similarly to the results reported by [34], GBF-1 and SEC-16 partially overlap by confocal microscopy (Fig. 2B), indicating that the cis-Golgi and the ERES are in very close proximity. To corroborate our results, we performed triple labeling of SEC-16, GBF-1 and UGTP-1 (Fig. 2C). This experiment demonstrates that GBF-1 is present on the cis-face of the Golgi at so-called transitional ER-Golgi elements [34], [39]–[41].

Next, we checked whether loss of GBF-1 would also cause Golgi organization to be affected. *gbf-1(RNAi)* caused the small Golgi foci to aggregate into large structures in at least 80% of the analyzed gonads, mainly around the nuclear membrane and cell-periphery (Fig. 2D), similar to what has been observed previously for *agef-1(RNAi)* and *arf-1.2(RNAi)* [3] (Fig. 2D). To distinguish between cis- and trans-Golgi defects, we analyzed oocytes by electron microscopy (Fig. 2E). *agef-1(RNAi)* resulted in large inflated cisternae, consistent with a block in secretion at the trans-Golgi [3], while upon *gbf-1(RNAi)* numerous vesicles accumulated at the cis-Golgi, representing most likely ER-derived COPII vesicles. Thus, loss of AGEF-1 and GBF-1 affect Golgi morphology in a different manner. We conclude that GBF-1/PHI-34 is indeed the homologue of mammalian GBF1.

Because *agef-1(RNAi)* caused the Golgi cisternae to be inflated, we asked whether this effect could potentially impair the association of GBF-1 with the Golgi. However, we found that GBF-1 was recruited to the cis-Golgi independently of AGEF-1 (Fig. S2). The GBF-1 localization to the Golgi is consistent with a block of TGN exit and a block in maturation in *agef-1(RNAi)* oocytes.

### *gbf-1(RNAi)* impairs ER structure but does not affect SEC-16 localization

**Figure 3:**
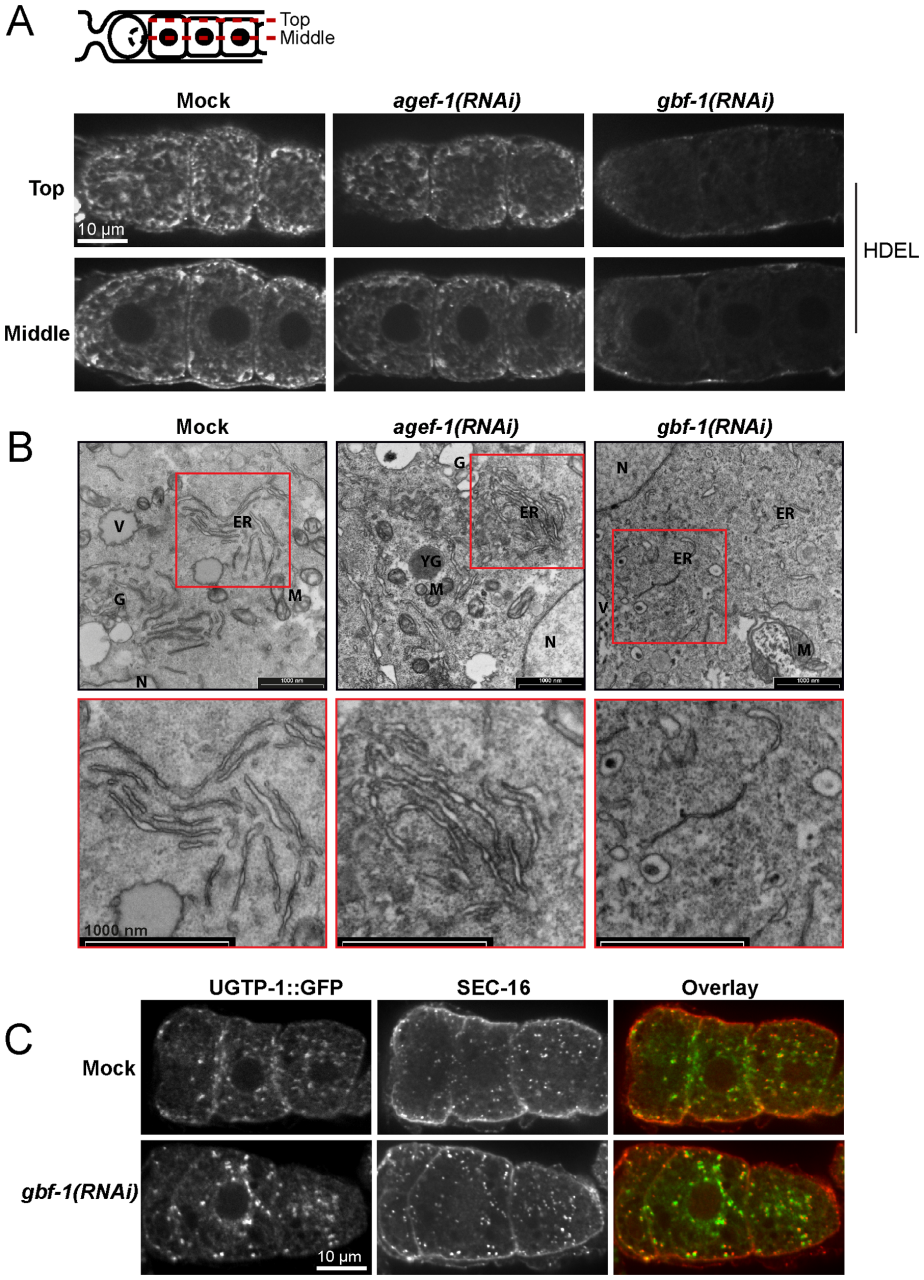
GBF-1 is required for ER integrity in oocytes. (A) *gbf-1(RNAi)* results in a disorganized ER in oocytes. The ER appeared dispersed in *gbf-1(RNAi)*. ER in the gonad was stained with an anti-HDEL antibody. Two focal planes are shown, one at the level of the nuclei (’Middle’) and one at the cell cortex (‘Top’) as indicated by the diagram. The spermatheca is located to the left. In wild type oocytes, the ER was organized in a weakly reticulated structure distributed throughout the cytoplasm. The ER morphology in *agef-1(RNAi)* oocytes was not affected, while in *gbf-1(RNAi)* oocytes the ER was present as dispersed haze. (B) TEM images of oocytes. The top panel shows a part of an oocyte and the bottom panel an enlargement as indicated by the red box. The ER in wild-type and *agef-1(RNAi)* oocytes was present in regular reticulate structures, indicated by ‘ER’. In *gbf-1(RNAi)* the ER was disorganized with regions where the lumen was collapsed. V, vesicle; M, mitochondria; G, Golgi; N, nucleus. (C) ERES are formed normally in *gbf-1(RNAi)* oocytes. Immunofluorescence images of oocytes which were stained with UGTP::GFP and anti-SEC-16. In a blind experiment, the SEC-16 staining in *gbf-1(RNAi)* and mock treated oocytes was indistinguishable from each other. Pictures refer to a single confocal plane. The spermatheca is located to the left. (A–C) The experiments have been performed at least three times using different RNAi feeding batches under the same experimental conditions. The selected images are representative for the observed phenotype.

Since GBF-1 localized in t-ER-Golgi elements and because *gbf-1(RNAi)* oocytes accumulated vesicles at the ER-Golgi interface, we asked whether loss of GBF-1 would also affect ER morphology. We stained the ER with antibodies against the HDEL ER-retention sequence [20]. In mature *C. elegans* oocytes, the ER is normally organized in a reticular, net-like structure throughout the cell (Fig. 3A) [20]. In *gbf-1(RNAi)* oocytes we observed a severely disturbed ER organization. In 50–60% of the gonads only a diffuse cytoplasmic signal was observed, while in another 30% of the gonads an intermediate phenotype was present, in which only weak reticulation was maintained around the cortex. No apparent defects were visible in *agef-1(RNAi)* oocytes (Fig. 3A). We corroborated these results by electron microscopy and found that in *gbf-1(RNAi)* oocytes most of the ER lacked a distinguishable lumen. No such effects were observed for *agef-1(RNAi)* and wild-type control (Fig. 3B). Thus, GBF-1 is required for both, ER and Golgi, organization. Given the strong effect of *gbf-1(RNAi)* on ER morphology, we checked, whether ERES marked by SEC-16 would also be dispersed. However, in all analyzed oocytes SEC-16 still localized in marked patches on close to the Golgi, similar to its localization in wild-type or mock-treated animals (Fig. 3C). These data indicate that despite perturbations in ER structure, ERES could still be maintained. These results are also in agreement with the vesicle accumulation observed on the cis-side of the Golgi in *gbf-1(RNAi)* oocytes by electron microscopy (Fig. 2D).

### Partial *gbf-1* knockdown impairs secretion

GBF-1 should recruit ARF-1.2 to the Golgi and promote the formation of COPI-coated vesicles destined to the ER. However, retrograde transport in the ER-Golgi shuttle is not easily studied, and in particularly not in a whole animal. In contrast, secretion is somewhat more easily assessed. Since a reduction of retrieval of transport factors from the Golgi to the ER will subsequently have some impact on secretion, we checked for secretion defects in *gbf-1(RNAi)* worms. As mentioned above, *gbf-1(RNAi)* worms have a weakened cuticle, which easily bursts, mostly through the vulva. The cuticle protects the worm from the outside and counteracts the turgor pressure. During the larval development, the old cuticle is shed and replaced several times, a process which is referred to as molting [42].

The cuticle is a collagenous extracellular matrix that is synthesized by an underlying hypodermis that surrounds the body of the animal. During synthesis, material is secreted from the apical membranes of the hypodermis [42]. Cuticle collagen secretion is highly dependent on the COPII pathway [43]. Therefore, a weakened cuticle can also be seen as a defect in secretion of cuticle components. In addition, we observed that *gbf-1(RNAi)* embryos were suffering from egg-shell defects (Fig. S3). We noticed that the embryos were not only osmosensitive (Fig. S3A), but in many cases did not even form a complete egg shell (Fig. S3B). Egg-shell defects are also a strong indication of secretion defects: immediately after fertilization of the oocyte, a wave of secretory granules stuffed with egg-shell components is secreted to form an eggshell and to protect the embryo from its environment [44], [45]. Failure to secrete these components efficiently will cause a defective egg shell that is permeable to small molecules.

**Figure 4:**
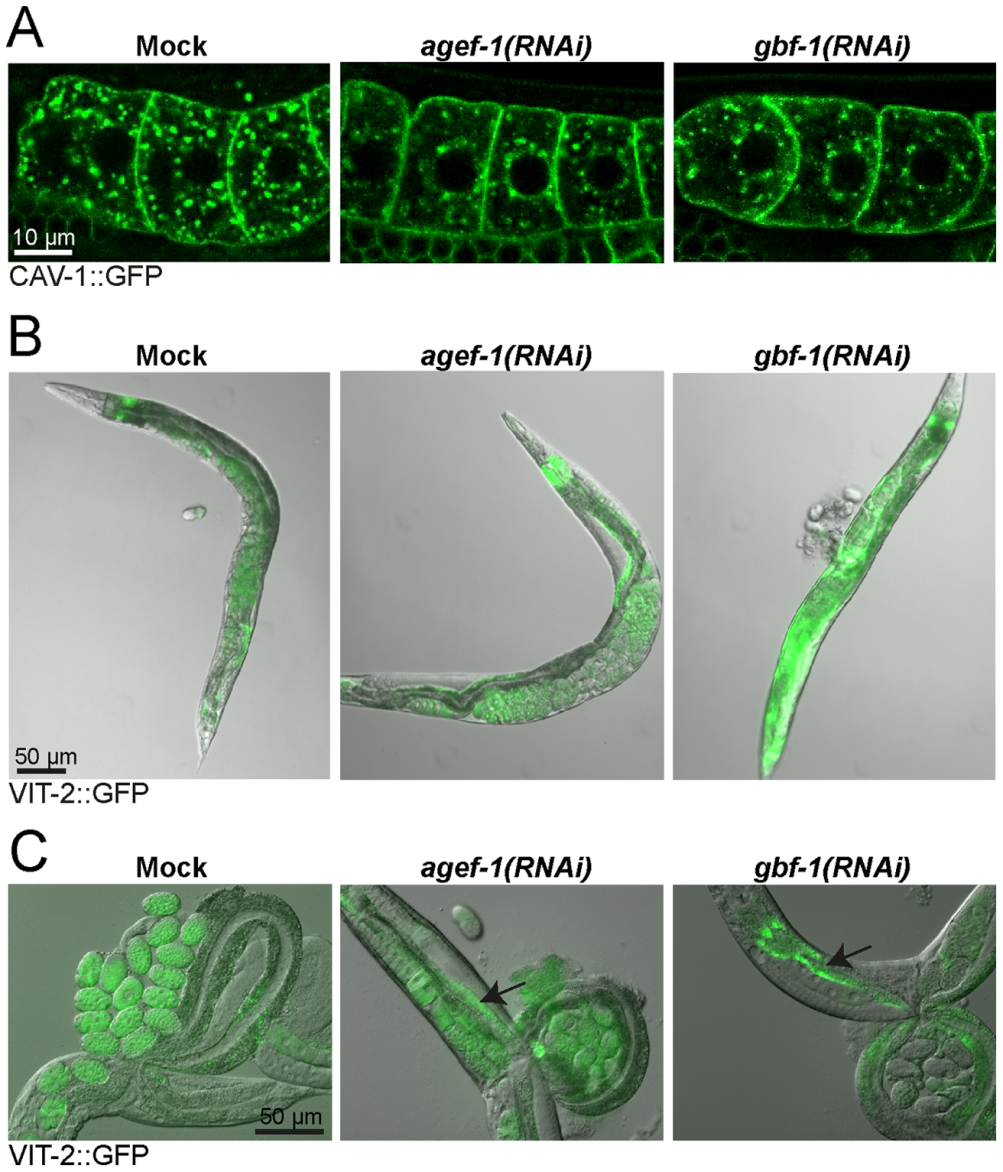
GBF-1 is required for secretion and endocytosis. (A) GBF-1 and AGEF-1 are required for caveolin secretion in oocytes. In both *agef-1(RNAi)* and *gbf-1(RNAi)* oocytes less CAV-1::GFP vesicles were observed throughout the cell when compared to mock treated animals. Single confocal planes are shown. (B) Endocytosis of yolk protein is impaired in *gbf-1(RNAi)* worms. In mock-treated and *agef-1(RNAi)* worms, YP170::GFP (VIT-2::GFP) accumulated in the maturing oocytes and in the developing embryos. In *gbf-1(RNAi)* worms a strong accumulation of YP170::GFP in the body cavity was observed in approximately 70% of the worms. (C) AGEF-1 and GBF-1 are required for efficient secretion of yolk protein. Both *agef-1(RNAi)* and *gbf-1(RNAi)* caused a buildup of yolk (YP170::GFP) in the intestine (indicated by black arrows). A DIC with GFP overlay is shown. (A–C) All experiments have been performed at least three times using similar experimental conditions using different RNAi feeding batches. The selected images are representative for the observed phenotype.

To get a more direct measure of defects in the secretory transport, we investigated caveolin (CAV-1) localization in oocytes. CAV-1 is found on cortical granules, and in mature oocytes CAV-1 is transported from the Golgi to the plasma membrane [19]. This process requires the action of AGEF-1 as well as ARF-1.2. In the absence of AGEF-1 or ARF-1.2, the internal CAV-1 structures become trapped in a perinuclear Golgi compartment [3]. Similar to the reported phenotype in *agef-1(RNAi)*, we also observed a comparable partial effect on CAV-1 secretion in *gbf-1(RNAi)* oocytes (Fig. 4A). To corroborate this partial secretion defect, we analyzed the secretion of yolk protein from gut cells. The yolk protein VIT-2 is synthesized in the gut, secreted into the body cavity and then taken up through receptor-mediated endocytosis into developing oocytes [23]. Both *agef-1(RNAi)* and *gbf-1(RNAi)* caused accumulation of yolk protein fused to GFP (VIT-2::GFP) in the intestine (Fig. 4B). Taken together, these data suggest, that GBF-1 plays a role in secretion. However, this role is likely indirect through GBF-1's involvement in retrograde transport to the ER.

### *gbf-1(RNAi)* causes a reduction in yolk uptake but not in yolk receptor recycling in oocytes

**Figure 5:**
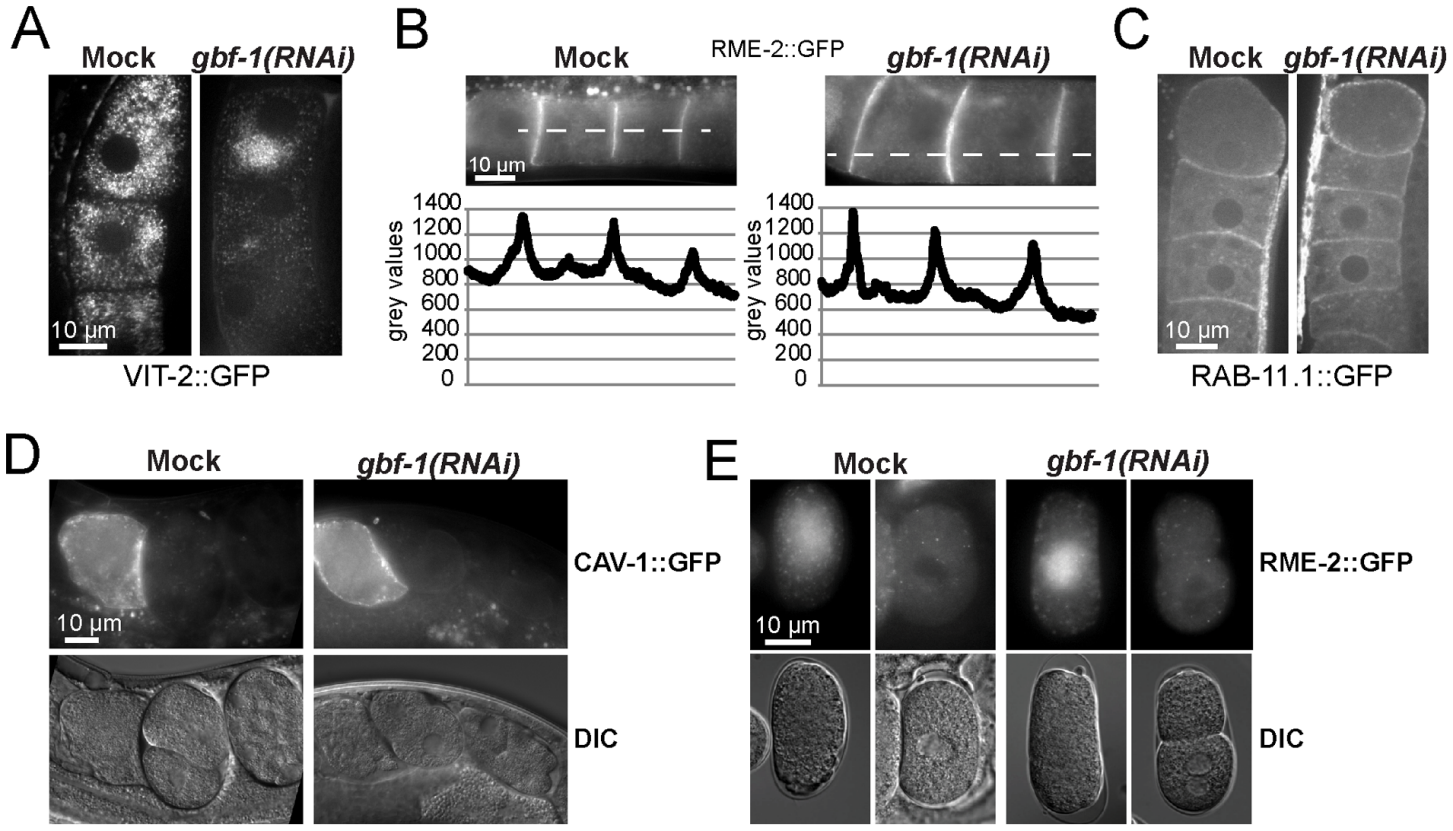
Recycling and internalization is not affected in *gbf-1(RNAi)* worms. (A) Yolk protein uptake is strongly reduced in *gbf-1(RNAi)* oocytes. A Single confocal plane through the center of the cells is shown. The spermatheca and the most mature oocyte are oriented toward the top. (B) Localization and intensity of the yolk receptor RME-2::GFP in *gbf-1(RNAi)* oocytes is undistinguishable from that in control worms. An intensity plot is shown below their corresponding images. The peaks represent the fluorescence at the plasma membrane between cell boundaries. Gray values are given in arbitrary units. (C) Recycling to the plasma membrane is unaffected in *gbf-1(RNAi)* oocytes. RAB11.1::GFP labeled recycling endosomes reached the plasma membrane normally in *gbf-1(RNAi)* oocytes compared to control oocytes. A single confocal plane is shown. (D) Knockdown of GBF-1 does not alter endocytosis or degradation of caveolin. Internalization of CAV-1::GFP in *gbf-1(RNAi)* oocytes was indistinguishable from that in mock RNAi worms. Spermatheca is located to the left. (E) RME-2 is endocytosed and degraded in *gbf-1(RNAi)* one-cell embryos. The time required for internalization of RME-2 in *gbf-1(RNAi)* oocytes seems not to be different from that observed in mock RNAi control worms. Anterior of the embryo is oriented towards the top. Corresponding fluorescence and DIC images are shown. (A–E) All experiments have been performed at least three times with comparable experimental conditions using different RNAi feeding batches. The selected images are representative for the observed phenotype.

When we analyzed the yolk secretion phenotype in *gbf-1(RNAi)* worms, we noticed in addition to yolk in the intestinal cells, a robust accumulation of yolk protein in the body cavity (Fig. 4B), indicative of a defect in receptor-mediated endocytosis in oocytes. Therefore, we decided to look more closely at the yolk content in oocytes in *gbf-1(RNAi)* worms and found that less VIT-2::GFP was taken up into the oocytes, but that the yolk granule size and distribution in the cell was not dramatically altered (Fig. 5A). We observed yolk accumulation in the body cavity robustly in more than 70% of the *gbf-1(RNAi)* worms that had been fed for 48 hour, whereas this was rarely seen in mock-treated animals (0–1%). The accumulation on yolk uptake was paralleled by a reduction of yolk content, although this was sometimes less pronounced than the accumulation phenotype. Since yolk production in the gut appeared not to be a problem, we wondered whether the yolk receptor RME-2 would not efficiently reach the plasma membrane in *gbf-1(RNAi)* oocytes. However, the RME-2::GFP levels at the plasma membrane were undistinguishable between mock treated and *gbf-1(RNAi)* oocytes (Fig. 5B). In addition, RAB-11.1, which is the Rab GTPase essential for recycling from endosomes to the plasma membrane was not affected by the reduction of GBF-1 (Fig. 5C). Therefore, the decrease in yolk uptake is not easily explained be a defect in receptor-mediated endocytosis. We cannot exclude that the kinetics of endocytosis and/or recycling of RME-2 is slightly reduced, which could account for the decrease in yolk content. Since the yolk granules appeared normal in size and distribution, endosomal delivery to the yolk granules in oocytes might also be largely independent of GBF-1.

Yolk granules may represent a special type of storage lysosomes [46] [47]. To investigate further whether endosomal delivery to lysosomes would require GBF-1 function, we investigated the fate of the yolk receptor after fertilization of the oocytes. In wild-type one-cell embryos, RME-2 is no longer required and therefore quickly endocytosed and subsequently degraded in the lysosome (Fig. 5D) [2]. No delay in RME-2 degradation was observed in *gbf-1(RNAi)* embryos when compared to mock RNAi treated embryos. Another protein, which is degraded in a similar time frame, is CAV-1 (Fig. 5E) [2], [3], and again, we did not detect any distinguishable delay in the degradation of the protein when GBF-1 levels were reduced. Therefore, our data suggest that endocytic trafficking to lysosomes in oocytes/one-cell embryos does not require GBF-1 function.

### The endosomal system is affected in the intestine in *gbf-1(RNAi)* animals

**Figure 6:**
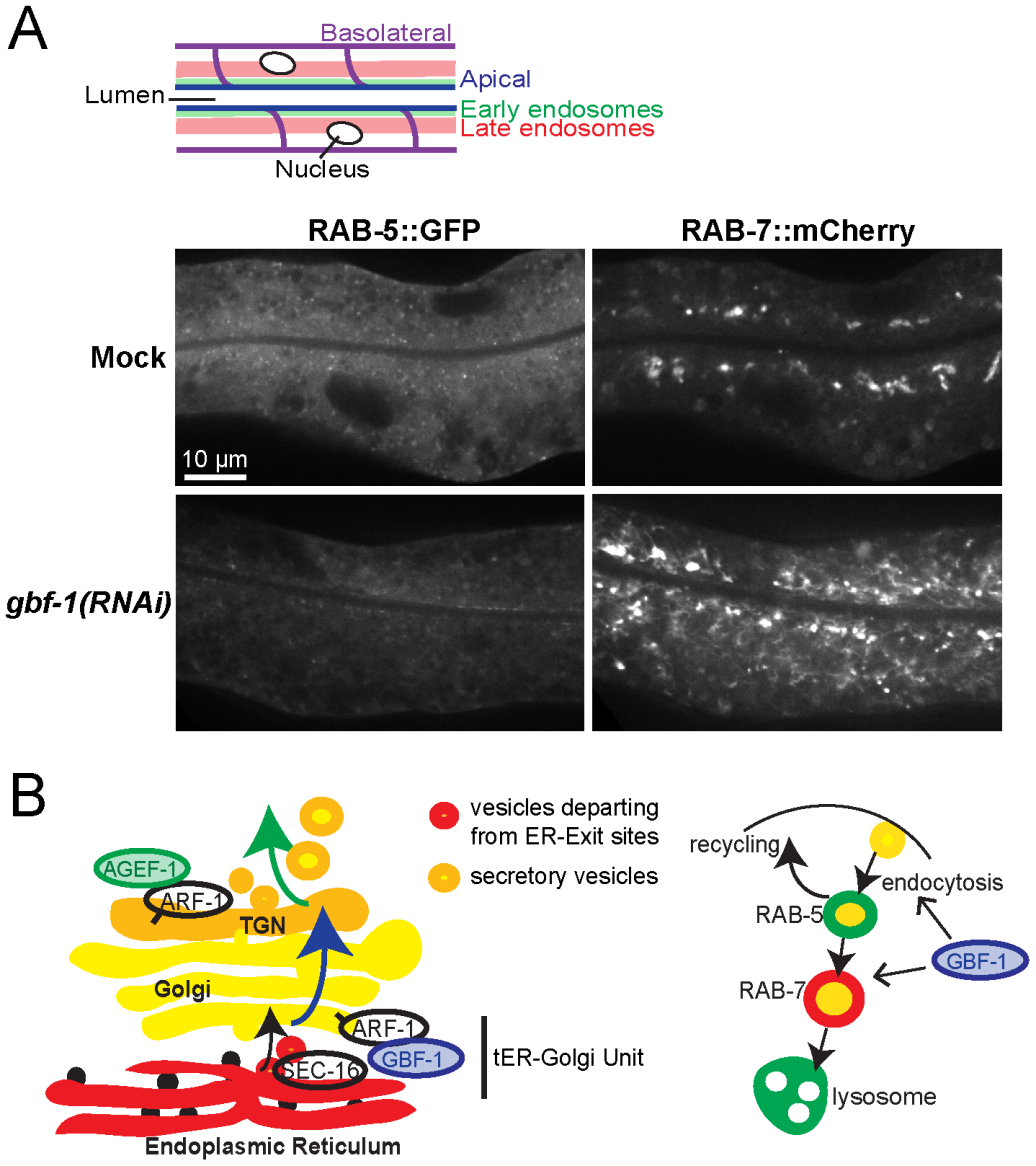
GBF-1 plays a role in later stages of endocytosis. (A) The endocytic pathway in the *gbf-1(RNAi)* intestine is severely disturbed. A schematic drawing of the intestine is shown. The expression of the early endocytic marker RAB-5::GFP was strongly reduced, while late endosomes appeared to form large aggregates as indicated by the presence of RAB-7. These phenotypes were observed in approximately 60%–90% of *gbf-1(RNAi)* worms compared to ≥10% in mock treated worms. The experiment was performed four times with 15 worms in each experiment. Single plane confocal images are displayed. (B) GBF-1 and AGEF-1 are both required for correct secretion from the Golgi. GBF-1 mainly acts at the cis-Golgi, while AGEF-1 is more important at the trans-Golgi. GBF-1 is involved in the endocytic pathway in *C. elegans*. Our data suggest a function at early and late endosomes, while in Drosophila GBF-1 was required at the plasma membrane.

To corroborate a role for GBF-1 in endocytosis, we created an integrated worm line that co-expresses RAB-5::GFP and RAB-7::mCherry in the intestine. In wild-type animals, RAB-5::GFP was enriched underneath the apical surface lining the gut lumen, while RAB-7::mCherry stains structures a bit further towards the cell center. In addition both markers label smaller endosomal structures throughout the cells (Fig. 6A). *gbf-1(RNAi)* caused a marked reduction of the RAB-5 signal (penetrance ranging from 50% to 85% of the worms in independent experiments). The lower RAB-5 signal in *gbf-1(RNAi)* may be due to a reduction of the number of early endosomes, or impaired endosomal recruitment of RAB-5, which could consequently lead to its degradation. Alternatively, *gbf-1(RNAi)* may impair expression of RAB-5::GFP. More importantly, RAB-7 was present on large interconnected and tubular endosomal structures throughout the cell, which were much brighter and larger than in mock-treated animals. This bright tubular network was found in 60% to 90% of the *gbf-1(RNAi)* animals, depending on the experiment, while in mock-treated animals only up to 10% of animals showed any networks. These data suggest that GBF-1 might be required for maintenance of endosome morphology and potentially for efficient endocytic transport in the gut epithelium. Since this effect was more prominent in the intestine than in oocytes, our data could indicate a tissue-specific requirement for GBF-1.

## Discussion

In this paper, we characterized the function of the ArfGEF GBF-1 in *C. elegans*. We found that GBF-1 performs evolutionary conserved functions of GBF1/garz/Gea1-2 ArfGEF guanine nucleotide exchange factors, namely a role in retrograde transport from the Golgi to the ER, in Golgi maintenance and secretion.

In addition, we found that loss of GBF-1, but not of AGEF-1 affects ER morphology. The mechanism by which GBF-1 helps to maintain ER morphology remains to be established. Given the evolutionary conserved established role of GBF-1 in retrograde transport from the Golgi to the ER, we would like to suggest that this effect is indirect and probably coupled through a decreased reflux of lipids. Strikingly, the lumen of the ER was very much reduced, suggesting a role in ER maintenance independent of the reduced secretion capacity from ER exit sites. In case of decreased secretion, cargo accumulation should occur and which would lead rather to a dilated ER. However, the secretory capacity in a non-fertilized oocyte might be rather low and therefore we may not observe dilated ER. Despite the strong morphological defects of the ER, the ER appears to be at least partial functional because the number and appearance of ERES was virtually unchanged and cargo such as CAV-1 was retained in Golgi membranes and not in the ER.

The ER phenotype, we observed for *gbf-1(RNAi)* was different to the one reported for *arf-1(RNAi)*, in which the ER formed clumps and was unable to cycle [20]. The reason for this could be twofold, on one hand, the *arf-1(RNAi)* ER phenotype was recorded in fertilized one-cell embryos, in which the ER cycles between a reticulate and sheet state in a cell-cycle dependent manner, while in this report we analyzed the ER in non-fertilized oocytes, in which the ER does not cycle between different states. On the other hand, as in mammalian cells [48] Arf4 and Arf5 GTPases may also be involved in retrograde transport from the Golgi to the ER and which would presumably also be activated by GBF-1.

We also noticed a secretion defect in *gbf-1(RNAi)* manifested by CAV-1 being present in internal structures around the nucleus, osmosensitive embryos, a weakened cuticle and accumulation of yolk protein-GFP in intestinal epithelial cells. Since *gbf-1(RNAi)* significantly perturbed Golgi morphology, this secretory defect might be secondary to the loss of Golgi integrity.

*gbf-1* knockdown also caused a defect in endocytosis. This may not be so surprising to initially because Gupta et al. [16] have shown that GBF1/garz activates Arf1 at the plasma membrane to allow endocytosis of GPI-anchored proteins through the GEEC pathway. However, we found a reduction in yolk protein uptake, which is a receptor-mediated and clathrin-dependent pathway [23]. Perhaps there is some cross-talk between these pathways as the yolk-depletion in the oocytes was not dramatic, and recycling of the yolk receptor appeared to be normal. Alternatively, given that *gbf-1(RNAi)* interferes with Golgi morphology, yolk protein synthesized in the gut epithelium may not have been glycosylated properly and therefore may bind with a somewhat lower affinity to the yolk receptor. We favor the second scenario, because we did not detect a defect in endocytosis and degradation of the yolk receptor or caveolin after fertilization of oocytes.

It is conceivable that different tissues and cell-types may differ in their requirements in the endocytic pathway, which may or may not be linked to the cargo load. Such differences might be reflected in oocytes versus gut epithelial cells. Consistent with this hypothesis, we found that *gbf-1(RNAi)* strongly affected RAB-7 positive endosomes and to a lesser extent early RAB-5 positive endosomes in the gut epithelium. Arf1 association with endosomes and its involvement in trafficking to lysosomes has been reported previously [46], [49], [50]. Recently, AGEF-1 has been implicated to play a role in lysosome morphology in coelomocytes [51]. Whether AGEF-1 would also act on endosomes remains unclear. Thus one possibility is that GBF-1 would be required on early/late endosomes and AGEF-1 on lysosomes. Another possibility is that both ArfGEFs have partial overlapping functions in endosomal transport. Such partial redundancy is not unheard of in case of ArfGEFs: the yeast ArfGEFs Gea1 and Gea2 have overlapping functions in retrograde transport from the Golgi to the ER [5]. A third possibility is that AGEF-1 and GBF-1 are present on the same compartment yet performing different functions. Again in yeast, Gea2 and Sec7 are associated with the TGN performing distinct functions [52], [53]. At present we cannot distinguish between these possibilities, and more studies are required to shed light on the specific functions of ArfGEFs.

## Supporting Information

Figure S1 **GBF-1 expression is significantly reduced by RNAi.** (A) Quantitative PCR of *gbf-1(RNAi)* and *agef-1(RNAi)* worms. The average of 5 independent experiments is shown by a bar graph. Standard deviation is indicated by the error bars. Mock RNAi control was normalized to 1. (B) Comparison of GBF-1 immunostaining in wild-type and *gbf-1(RNAi)* gonads to demonstrate antibody specificity. A strong reduction of signal was observed in *gbf-1(RNAi)* tissues compared to the mock RNAi control. Intestine and gonad are indicated. The experiment was performed multiple times, also comparing different feeding times ranging from 24 to 72 hours. In all cases the signal in *gbf-1(RNAi)* worms was clearly reduced in the gross majority of the gonads compared to mock treated animals. The selected images are representative for the knock-down efficiency at 48 hours. (C) A western blot of whole worm lysate. In *gbf-1(RNAi)* a band of the expected protein size of 220 kD is reduced compared to mock RNAi worms as indicated by the black arrow.
(TIF)

Figure S2 **GBF-1 localizes to the Golgi independent of AGEF-1.** In *agef-1(RNAi)* oocytes, the Golgi forms aggregates. GBF-1 was in each observed gonad robustly recruited to these Golgi aggregates. Single confocal planes at the cortex and the center of the cell of the most proximal oocytes are shown. An enlargement of the white box is shown on the right. The experiment was performed three times using the same conditions.
(TIF)

Figure S3 **GBF-1 is important for egg-shell secretion.** (A) *gbf-1(RNAi)* eggs are sensitive to the osmolarity of the environment. In a high salt buffer, the *gbf-1(RNAi)* embryos shrunk, whereas mock RNAied embryos were protected from their environment by an impermeable egg-shell. DIC pictures of two-cell stage embryos are shown. Cropped edges of the rotated image are indicated in black. (B) *gbf-1(RNAi)* embryos frequently lack completely the formation of a proper egg-shell. As a result the uterus of the *gbf-1(RNAi)* worms was filled with an amorphous mass of cells. DIC images are shown. (A–B) These phenotypes were consistently seen throughout all our different experiments.
(TIF)
