# Supplementary figures and images for: The ArfGEF GBF-1 Is Required for ER Structure, Secretion and Endocytic Transport in C. elegans
Source: PLoS One. 2013 Jun 19;8(6):e67076. doi: 10.1371/journal.pone.0067076 (PMC3686754; doi:10.1371/journal.pone.0067076)

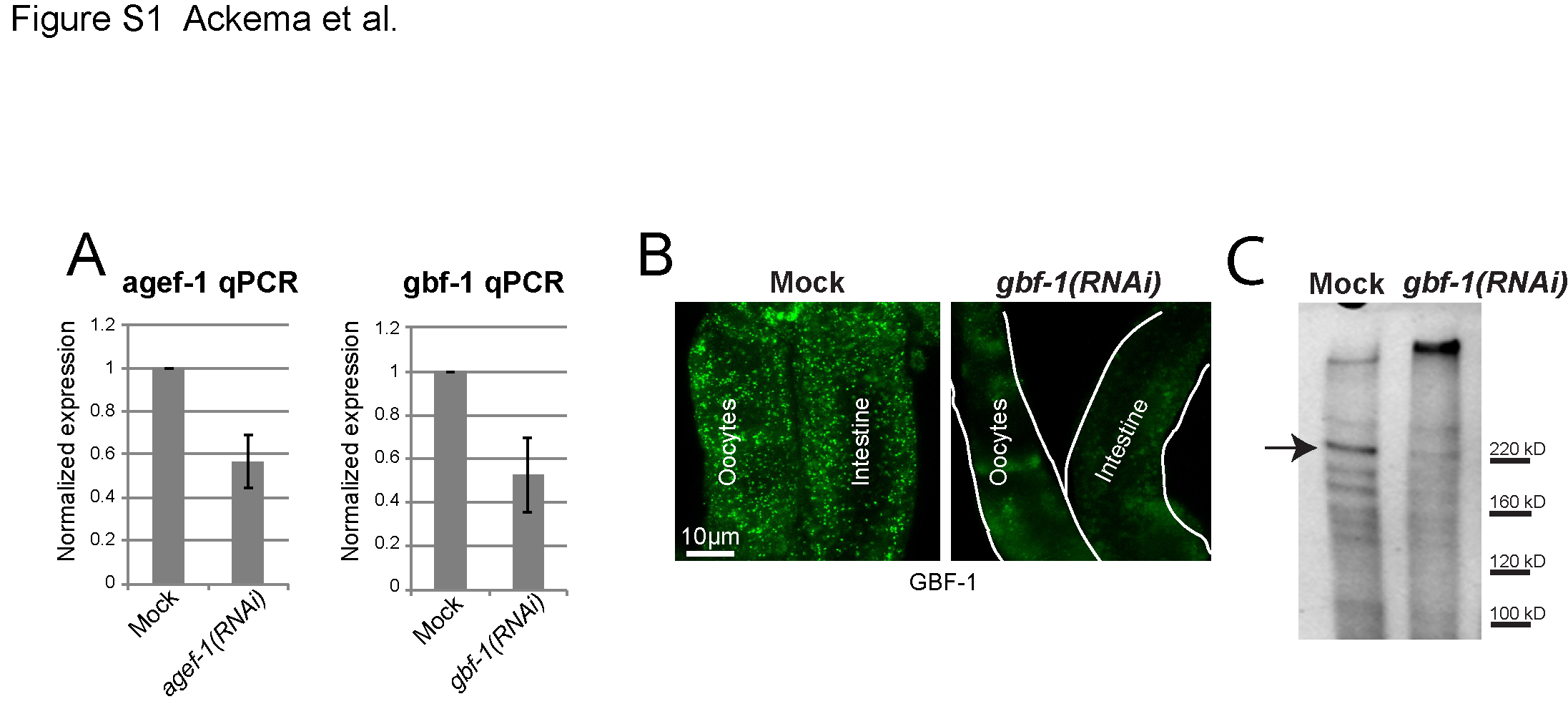

Supplement: Figure S1 — GBF-1 expression is significantly reduced by RNAi. (A) Quantitative PCR of gbf-1(RNAi) and agef-1(RNAi) worms. The average of 5 independent experiments is shown by a bar graph. Standard deviation is indicated by the error bars. Mock RNAi control was normalized to 1. (B) Comparison of GBF-1 immunostaining in wild-type and gbf-1(RNAi) gonads to demonstrate antibody specificity. A strong reduction of signal was observed in gbf-1(RNAi) tissues compared to the mock RNAi control. Intestine and gonad are indicated. The experiment was performed multiple times, also comparing different feeding times ranging from 24 to 72 hours. In all cases the signal in gbf-1(RNAi) worms was clearly reduced in the gross majority of the gonads compared to mock treated animals. The selected images are representative for the knock-down efficiency at 48 hours. (C) A western blot of whole worm lysate. In gbf-1(RNAi) a band of the expected protein size of 220 kD is reduced compared to mock RNAi worms as indicated by the black arrow. (TIF) [file pone.0067076.s001.tif]

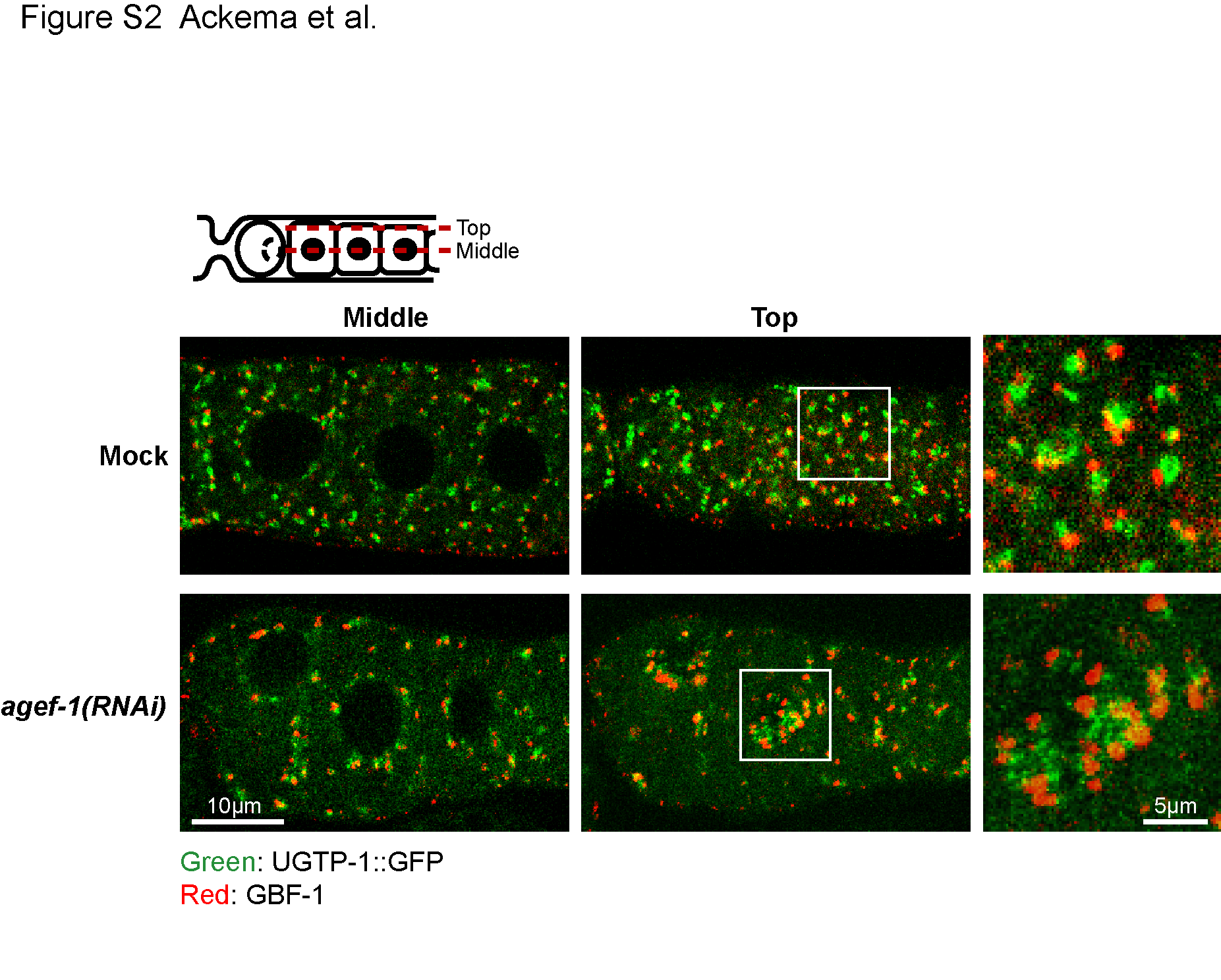

Supplement: Figure S2 — GBF-1 localizes to the Golgi independent of AGEF-1. In agef-1(RNAi) oocytes, the Golgi forms aggregates. GBF-1 was in each observed gonad robustly recruited to these Golgi aggregates. Single confocal planes at the cortex and the center of the cell of the most proximal oocytes are shown. An enlargement of the white box is shown on the right. The experiment was performed three times using the same conditions. (TIF) [file pone.0067076.s002.tif]

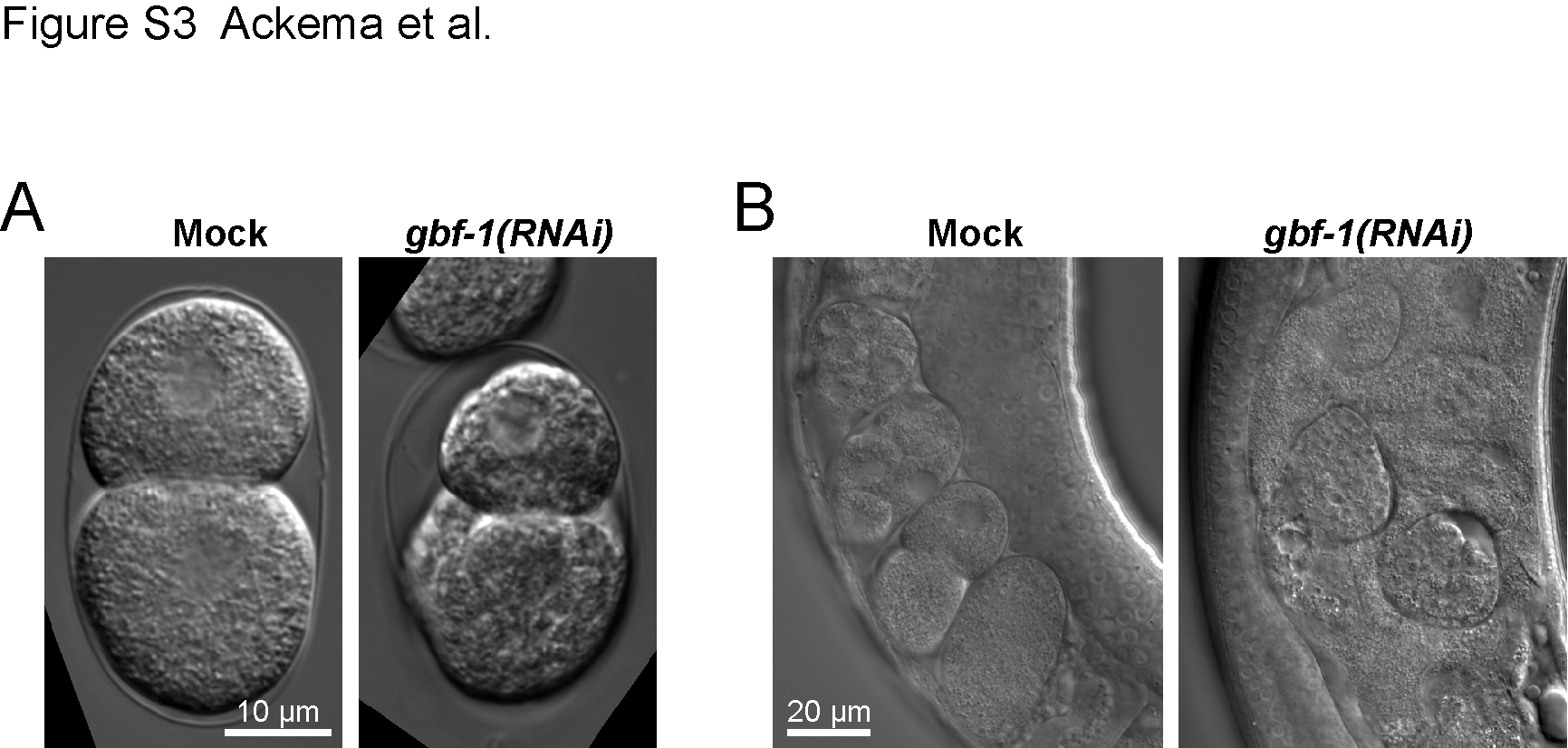

Supplement: Figure S3 — GBF-1 is important for egg-shell secretion. (A) gbf-1(RNAi) eggs are sensitive to the osmolarity of the environment. In a high salt buffer, the gbf-1(RNAi) embryos shrunk, whereas mock RNAied embryos were protected from their environment by an impermeable egg-shell. DIC pictures of two-cell stage embryos are shown. Cropped edges of the rotated image are indicated in black. (B) gbf-1(RNAi) embryos frequently lack completely the formation of a proper egg-shell. As a result the uterus of the gbf-1(RNAi) worms was filled with an amorphous mass of cells. DIC images are shown. (A–B) These phenotypes were consistently seen throughout all our different experiments. (TIF) [file pone.0067076.s003.tif]
